# Dairy farmers’ decision‐making to implement biosecurity measures: A study of psychosocial factors

**DOI:** 10.1111/tbed.13387

**Published:** 2019-11-02

**Authors:** Sebastián Moya, Francisco Tirado, Josep Espluga, Giovanna Ciaravino, Ramon Armengol, Javier Diéguez, Eduardo Yus, Bibiana Benavides, Jordi Casal, Alberto Allepuz

**Affiliations:** ^1^ Department of Animal Health and Anatomy Universitat Autònoma de Barcelona (UAB) Barcelona Spain; ^2^ Department of Social Psychology Universitat Autònoma de Barcelona (UAB) Barcelona Spain; ^3^ Departament of Sociology Universitat Autònoma de Barcelona (UAB) Barcelona Spain; ^4^ Department of Animal Science Universitat de Lleida (UDL) Lleida Spain; ^5^ Department of Anatomy, Animal Production and Veterinary Clinical Sciences Area of Animal Production Universidad de Santiago de Compostela (USC) Lugo Spain; ^6^ Department of Animal Pathology Area of Animal Health Universidad de Santiago de Compostela (USC) Lugo Spain; ^7^ Department of Animal Health Universidad de Nariño (UDENAR) Pasto Colombia; ^8^ Centre de Recerca en Sanitat Animal (CReSA) Institut de Recerca i Tecnologia Agroalimentàries (IRTA) Universitat Autònoma de Barcelona (UAB) Barcelona Spain

**Keywords:** biosecurity, dairy farms, disease prevention, epidemiology, qualitative, sociology

## Abstract

Biosecurity measures are a set of management procedures that prevent the risk of introducing and spreading infectious diseases to a farm, although these measures are rarely implemented in dairy farms. There are some studies that have identified that the decision to implement biosecurity measures can be influenced by several psychosocial factors (attitudes and behaviours). Thus, the objective of this study was to examine the psychosocial factors (and their interactions) influencing the implementation of biosecurity measures in dairy farms in Spain, through the views of dairy farmers and veterinarians from Catalonia (northeast Spain) and Galicia (northwest Spain). Face‐to‐face in‐depth interviews were performed with 16 dairy farmers (nine from Catalonia and seven from Galicia) and 16 veterinarians (eight from Catalonia and eight from Galicia). Grounded theory analysis was performed on the transcripts, following the subtopics of: information sources, individual factors of the farmer, social dynamics, official veterinary services and other factors. The study identified the importance of veterinarians as a source of information, including their communication skills, the individual experiences of farmers, traditions of the farms and availability of time and space in the dairy farmer's decisions making. Further, it suggests the need to deepen the knowledge of the farm workers and the obligatory biosecurity measures. This research represents a starting point to develop future strategies to improve the implementation of biosecurity measures in dairy farms.

## 1 INTRODUCTION

Biosecurity can be defined as the methods that are used to stop a disease or infection from spreading from one person, animal, or place, to others (Cambridge Dictionary, 2019). On farms, this concept is defined as a set of management procedures that prevent the risk of introducing disease agents into a farm (external biosecurity) and that minimize the spread of disease agents within the herd (internal biosecurity) (FAO, 2010).

The implementation of biosecurity measures can improve animal health (Oliveira, Sørensen, & Thomsen, 2017) and animal welfare (Barkema et al., 2015) and therefore increase productivity in dairy farms (Postma et al., 2016a). In addition, an association has been observed between higher biosecurity and a reduction in antibiotic use (Laanen et al., 2013; Postma et al., 2016b). Despite this, biosecurity measures in dairy farms are rarely implemented (Renault, Damiaans, et al., 2018a; Sahlström, Virtanen, Kyyrö, & Lyytikäinen, 2014; Sarrazin, Cay, Laureyns, & Dewulf, 2014).

The implementation of biosecurity measures at the farm level requires the adoption of a set of attitudes and behaviours by individuals. These attitudes and behaviours are within the so‐called psychosocial factors. Psychosocial factors refer to the combination of psychological (level of individual processes and meanings) and social (level of human society, social structure and social processes) factors. In this way, the psychological factors can mediate with the social factors, and the social factors can affect the individual factors (Stansfeld & Rasul, 2007).

Different studies have identified several psychosocial factors in dairy farmers and veterinarians that might influence their decision on whether or not to implement biosecurity measures. Among these factors, it has been described that the attitude of farmers and veterinarians towards the implementation of biosecurity measures might be affected by the technical knowledge they have (Frössling & Nöremark, 2016; García & Coelho, 2014; Toma, Low, Vosough, Matthews, & Stott, 2015), the individual experiences they have lived (Broughan et al., 2016), the importance they can attribute to risks (Renault, Humblet, et al., 2018b), and the benefits they can obtain from measures implemented (Ciaravino et al., 2017). Moreover, their behaviour towards the implementation of biosecurity measures has also been related to their perceived social pressure to apply these measures (i.e. the subjective norm (Ajzen, 1991)). This might be influenced by personal relationships (Cardwell et al., 2016; Ellis‐Iversen et al., 2010; Shortall et al., 2016), action and communication dynamics (Heffernan, Nielsen, Thomson, & Gunn, 2008; Sayers, Good, & Sayers, 2014), or by the relationship between farmers and veterinarians working in the public administration (i.e. official veterinary services (OVS)), where organizational and institutional support (Kristensen & Jakobsen, 2011) and bureaucracy (Hovi, Mcleod, & Gunn, 2005) can be relevant. And finally, their behaviour can be affected by individual factors such as age and gender (Frössling & Nöremark, 2016) or location and size of the farm (Hoe & Ruegg, 2006; Sayers et al., 2013), which may influence their willingness to invest in biosecurity measures (Gunn, Heffernan, Hall, McLeod, & Hovi, 2008). Time and economic constraints may also be relevant (Brennan & Christley, 2012; Pritchard, Wapenaar, & Brennan, 2015), as well as incentives (Frössling & Nöremark, 2016), access to information sources (Laanen et al., 2014; Toma, Stott, Heffernan, Ringrose, & Gunn, 2013), education, and awareness (Brennan & Christley, 2012; Kuster, Cousin, Jemmi, Schüpbach‐Regula, & Magouras, 2015).

**Figure 1:**
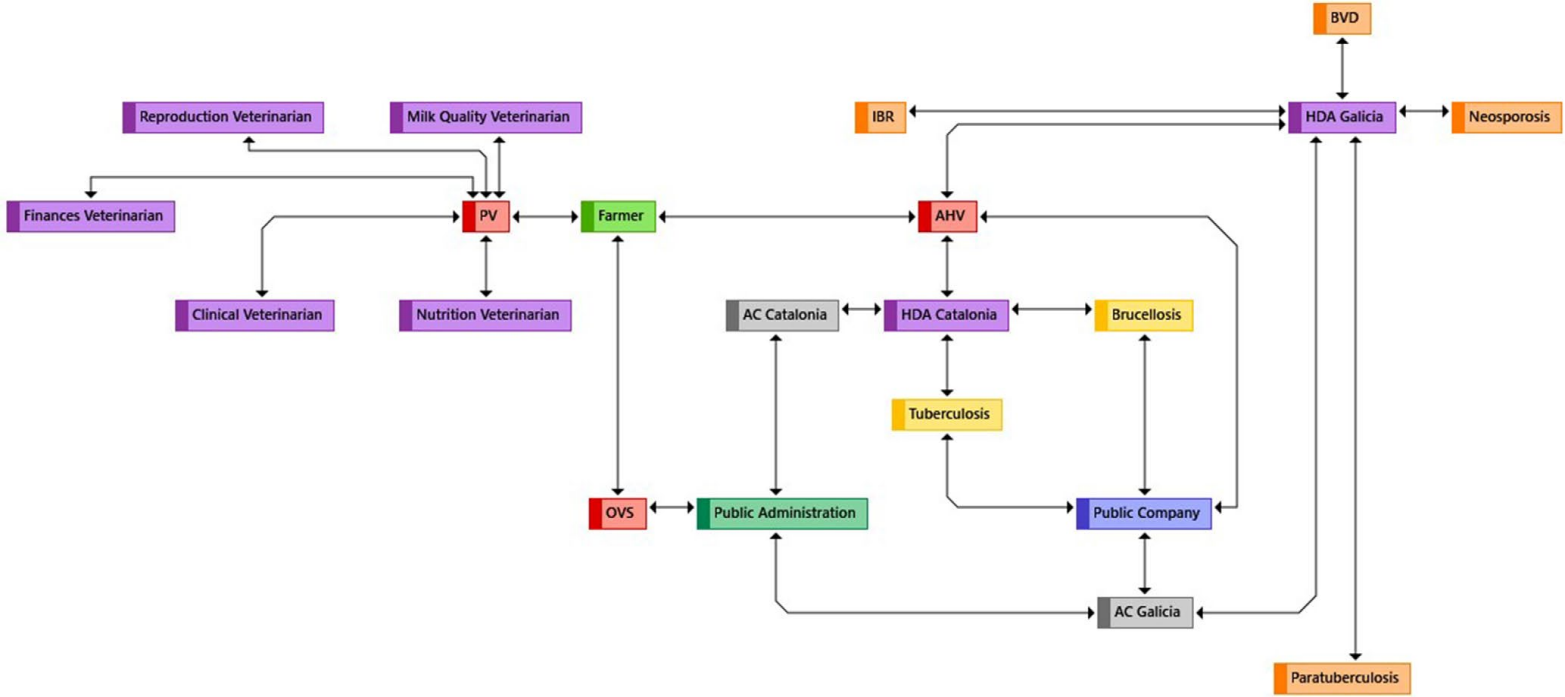
Interaction among veterinary profiles. Scheme obtained from ATLAS.ti 8.2.34 through the codes. AC: Autonomous Community; AHV: animal health veterinarians; HDA: health defence association; OVS: official veterinary services; PV: private veterinarians

In Spain, there are several profiles of dairy farmers and veterinarians. On one hand, there are conventional and organic farms, which differ mainly in that the latter have a holistic and integral approach (self‐sufficiency) (Stonehouse, Clark, & Ogini, 2001) and must adhere to strict standards with regard to the use of agricultural chemicals (such as synthetic fertilizers and pesticides) and animal medicines (such as antibiotics, anti‐parasitics and hormones; EC, 2019). On the other hand, there are private veterinarians (PV), animal health veterinarians (AHV) and OVS (Figure 1). PV are the technical advisors who are hired and paid by the dairy farmer for different areas (e.g. clinical, reproduction, milk quality or nutrition, among others). AHV fall in two main groups: (a) health defence association (HDA) veterinarians. HDA are constituted by farmers associations that aim to improve the health status of their herds, but the responsibilities of the contracted HDA can vary among regions. For example, in northwest Spain (i.e. Galicia) they are only involved in voluntary control programmes of non‐regulated diseases (such as Infectious Bovine Rhinotracheitis (IBR), Bovine Virus Diarrhoea (BVD), paratuberculosis or neosporosis). Contrary, in northeast Spain (i.e. Catalonia) these veterinarians are just involved in control programmes of regulated diseases (such as tuberculosis or brucellosis). Nevertheless, in both cases, regardless their involvement with regulated or non‐regulated diseases, the HDA are recognized by the public administration and regulated according with national legislation (Royal Decree 842/2011). These are hired by the farmer association itself through the payment of a quota. And, although these associations can receive public funds for the development of these programs, these are not linked to public administration. And (b) veterinarians who carried out mandatory eradication programmes (i.e. regulated diseases) contracted by the OVS (i.e. public administration). They carry out the fieldwork of these programmes and provide all the data to the public administration. As a matter of fact, in Galicia, the control of tuberculosis and brucellosis is exclusively carried out by these veterinarians. In the case of Catalonia, on the contrary, just one HDA is responsible for the mandatory eradication programmes. Thus, in this area there are no specific entities charge of the control of non‐regulated diseases. Finally, OVS monitor farms in various fields, such as animal health. The objective of this monitoring is for farmers to carry out certain management that are under direct or indirect official legal frameworks.

To improve biosecurity, it is necessary to identify the psychosocial factors (and their interactions) that can influence the decision for the implementation of biosecurity measures. Thus, an understanding of each of them and their interactions might allow establishing the individual and collective processes that would be necessary to improve the implementation of biosecurity measures on dairy farms. Therefore, the aim of this study was to explore the psychosocial factors of dairy farmers and veterinarians that determine the implementation of biosecurity measures in dairy farms in Spain. The study results could lead to providing recommendations to improve biosecurity in dairy farms. [Colour figure can be viewed at http://wileyonlinelibrary.com]

## 2 MATERIALS AND METHODS

### 2.1 Area of study

The present study was carried out in two Autonomous Communities of Spain, Catalonia (northeast) and Galicia (northwest), which contain 11% and 38% of dairy cattle population, respectively (MAPAMA, 2019a), with a high level of dairy productivity, 66,270 and 231,331 tons per year, respectively (FEGA, 2019). However, the type of farms in both areas are very different, while in Catalonia the dairy farms have a medium–large size (240–890 lactating cows per farm), in Galicia they are smaller (33–73 lactating cows per farm) (MAPAMA, 2019b) and they have been developed around homes, being small family farms in most cases (De Llano, 1989).

### 2.2 Study design

A qualitative research design was used in this study using individual in‐depth interviews. These interviews were conducted with dairy farmers and veterinarians from both Autonomous Communities. Participants were selected by intentional sampling to identify different discourses through maximum variation (Flick, 2014).

### 2.3 In‐depth interviews

For the in‐depth interviews, a thematic guide was produced based mainly on scientific articles related to psychosocial factors in dairy farms. Subsequently, modifications were made based on the different views of the research group, and final corrections were made based on a pilot interview with a dairy farmer. In this way, a thematic guide was obtained composed of five topics: (a) knowledge; (b) direct actions; (c) sources of information; (d) experiences; and (e) expectations (Annex). The questions asked to the veterinarians were in relation to the dairy farmers’ attitudes and behaviours.

The semi‐structured in‐depth interviews were conducted face‐to‐face and tape‐recorded. The interviews were conducted between 16 February and 19 July 2018 in Catalonia, and between 3 July and 12 July 2018 in Galicia.

A total of 32 participants were interviewed. Different profiles of dairy farmers and veterinarians were considered in order to have different views (Table 1). Only the profiles of PV and AHV were considered, but not OVS. However, for results and analysis, these profiles were unified only in farmers and veterinarians.

**Table 1:** Profiles of dairy farmers and veterinarians that participated in the present study

Dairy farmers profile	Catalonia	Galicia	Veterinarians profile	Catalonia	Galicia
Conventional	7	6	Clinical (PV)	2	1
			Reproduction (PV)	2	1
			Milk Quality (PV)	0	2
Organic	2	1	Nutrition (PV)	0	1
			Finances (PV)	2	1
			AHV	2	2
Total (*)	9 (3*)	7 (1*)	Total	8 (2*)	8 (3*)

Note

Abbreviations: AHV, animal health veterinarians, PV, private veterinarians. *In brackets, number of women participating.

Each interview lasted between 45–90 min. In the first minutes of the interview, general questions were asked to generate a relaxed atmosphere between the interviewee and the interviewer. These questions were related to personal and professional topics, showing interest in knowing their answers. In the following minutes, in‐depth questions were asked. These questions were directly related to the topics of the thematic guide. In the following minutes, corroborative questions were asked to answer the generated doubts. These questions were related to their answers to the previous questions.

The audios of the in‐depth interviews were reviewed and subsequently transcribed to analyse their data. In the transcripts, the participants were labelled with an initial letter ‘F’ for dairy farmers or ‘V’ for veterinarians, followed by a ‘C’ for the people in Catalonia or a ‘G’ for Galicia, with a final numbering from 1 to 9 for their differentiation (e.g. FC1 refers to a farmer in Catalonia).

### 2.4 Analysis of data

The data collected (answers of the participants) were analysed using ATLAS.ti 8.2.34, a software based on grounded theory. Grounded theory is a method of interpretative analysis that allows developing a theory that includes social processes and specific concepts (Tesch, 1990; Trinidad, Carrero, & Soriano, 2006). This method is based on constant comparative processes, theoretical criteria and conceptual saturations to provide explanations and important applications (Glaser & Strauss, 1967; Trinidad et al., 2006).

Throughout the discourses of participants, the software allowed us to recognize a set of segments of information that were of interest for the research objectives (i.e. codes, also called concepts or categories). Moreover, it allowed to generate a set of stand‐alone ideas based on these discourses for the researchers themselves (i.e. memos) (ATLAS.ti, 2019). In this way, the software introduced the discourses of participants as citations, which were associated to codes (codes groups), and memos.

The previous results were then sent via e‐mail to the participants so that they could provide some feedback. Thus, this feedback was taken into account when interpreting the results of the present study.

## 3 RESULTS

**Figure 2:**
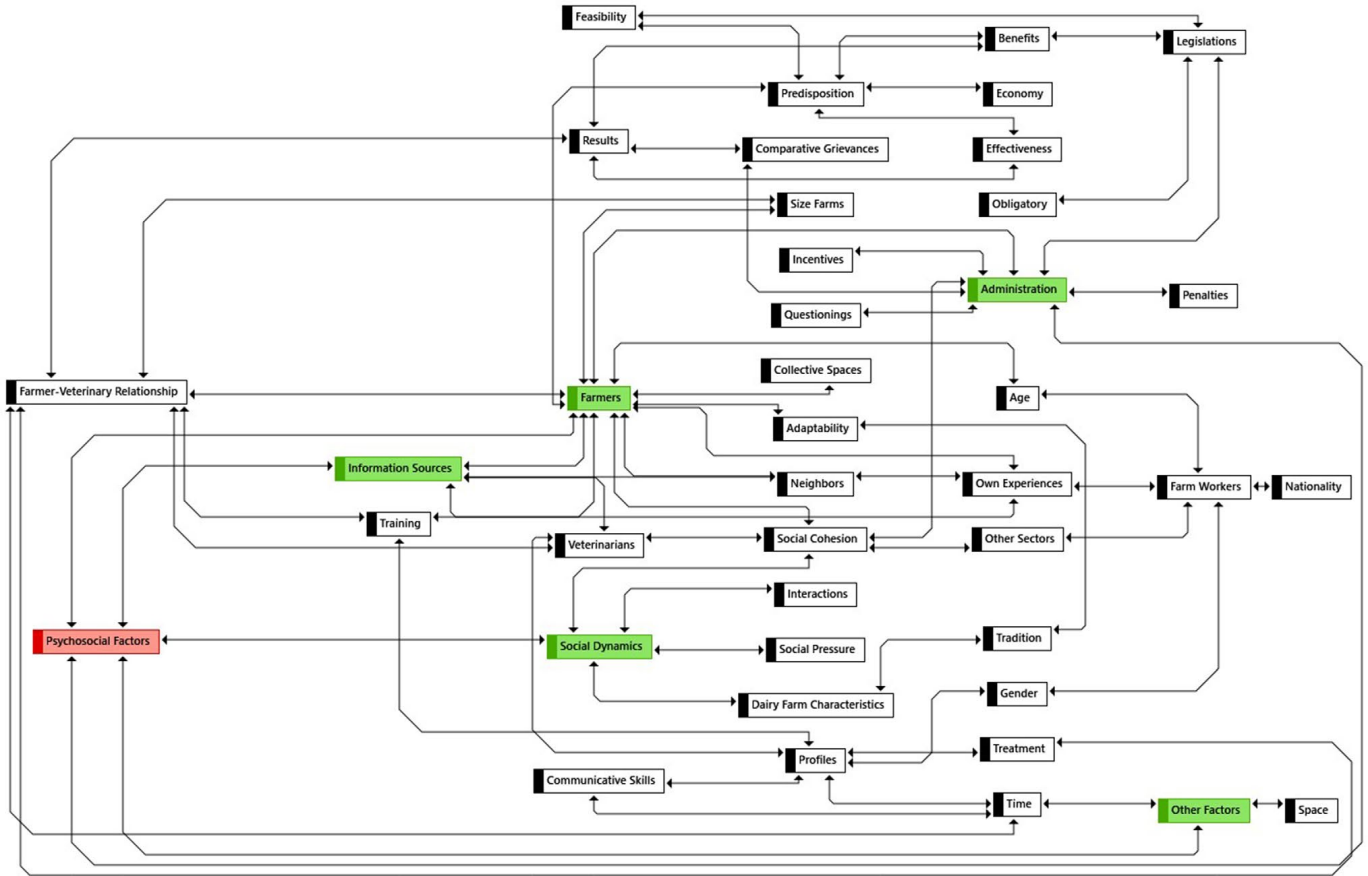
Interaction among the various psychosocial factors. Scheme obtained from ATLAS.ti 8.2.34 through the codes [Colour figure can be viewed at http://wileyonlinelibrary.com]

A total of 178 citations, 39 codes (nine codes groups) and 25 memos were selected and used for the final analysis process. These citations are in their original language (Spanish) in the Annex to the present study. Comparatively, these citations were the most heterogeneous of all. The citations were organized following the subtopics of: (a) information sources; (b) individual factors of farmer (i.e. internal world of the farmer); (c) social dynamics (internal and external)); (d) official veterinary services (OVS, bad policemen or necessary enemies?); and (e) other factors (variables of time and space). Thus, attitudes and behaviours and their diverse interactions are intertwined with the five topics of the thematic guide. In this way, several psychosocial factors influencing the application of biosecurity measures in dairy farms were mentioned during the interviews, which can interact with each other in different ways, as is shown in Figure 2.

### 3.1 Information sources

The interviewees indicated that farmers can use different sources of information to learn about biosecurity, but they pointed out veterinarians and other farmers as the most relevant sources.

The farmers emphasized that veterinarians know the farms in more detail and, therefore, have a greater capacity to influence the decision to apply biosecurity measures by insisting and persisting on the possible risks to which the farms are exposed. Veterinarians suggest options to the farmers that may be viable depending on the priorities that the farms have. These suggestions, in the opinion of veterinarians, are given spontaneously or as a result of a direct consultation, since they do not want their farmers to believe that they have a conflict of interest.

The interviewed also commented that the veterinarian profile can influence advice on biosecurity, for example, the HDA veterinarians. This veterinarian profile advises on biosecurity and raises awareness directly or indirectly about these measures in their daily practice. In addition, the voluntary membership to an HDA by dairy farms was linked to an improvement in the sanitary status of the farm due to a greater biosecurity awareness:

FG2: *"(...) Many workshops in the HDA also helps. In diarrhoea of small calves (...) it has helped us a lot (...), deaths are reduced (...). The HDA insist a lot that we are going to do this and such, little by little, but it insists on many things. He/she is a good technician (...), very involved (...)" [Original: “(…) Ayuda también muchos talleres en la ADS. En temas de diarreas de terneros pequeños (…) nos ha ayudado mucho (…), se reducen las muertes (…). La ADS insiste mucho que vamos a hacer esto y tal, poco a poco, pero insiste en muchas cosas. Es un buen técnico (…), muy implicado (…)”]*

VG2: *"(...) I had two important outbreaks of IBR (...), one of almost 500 heads, and another of about 100 (...). Since we have been working in HDA, the numbers have been decreasing (...). We, those of HDA, are those who do the 90% of biosecurity work (...)" [Original: “(…) Tuve dos brotes importantes de IBR (…), uno de casi 500 cabezas, y otro de unas 100 (…). Desde que llevamos trabajando en ADS, los números fueron disminuyendo (…). Somos los de ADS los que hacemos el trabajo de bioseguridad en el 90% (…)”]*

In relation to the veterinarian's ability to influence the farmer's decision to apply biosecurity measures, an important factor that emerged in the interviews was the existing trust relationship between the farmer and the veterinarian. This relationship, in the opinion of the interviewees, is influenced by the time and treatment given–received, which in turn is influenced by the profile of the veterinarians and their level of training and communication skills. In the interviews, different types of relationships were described. One of them was described as ‘*close*’ and was characterized by the long periods of time farmer–veterinarian have worked together. In this type of relationship both farmers and veterinarians feel heard and, therefore, can agree on their decisions. However, other relationships were also described that were ‘*more distant*’ due to the limited periods of time they share, such as with the AHV from public animal health companies of only a few hours per year, different from those of clinical, reproduction, or nutrition. In the same way, in the interviews, ‘*close*’ relationships were also described to be characterized by a friendly treatment (i.e. due to the dynamics of nearby social circles). In these dynamics, there may be interactions that involve personal areas with reciprocal understandings and decisions mutually agreed directly or indirectly. These relationships were linked to the size of the farms (more on small ones), and the results that farmers can observe regarding the advice given by the veterinarian. It was also mentioned that a friendly treatment could lead to the farmers ignoring a mistake by the veterinarians, unlike an unfriendly treatment. However, there were opposing opinions among veterinarians. Some of them were in favour of a ‘*close’* relationship with the farmer, involving personal and professional aspects, while others preferred a purely professional relationship in order to avoid conflicts of interest.

The ability of the veterinarian to influence the farmer's decision to apply biosecurity measures was also linked to their level of training in biosecurity and their communication skills. Some veterinarians expressed that they did not have enough arguments to defend the application of biosecurity measures and that they required extra training to transfer biosecurity to dairy farms. Similarly, it was mentioned that there was a lack of persuasive skills for farmers to implement such measures due to the lack of observable benefits attributable to the implementation of biosecurity measures in the short term. In addition, veterinarians mentioned that farmers require time to come to terms with the proposals, with communication skill being a key factor to avoid fatigue by the farmers due to the insistence of the veterinarians:

VC4: *"(...) I do not know if we can convince them enough, because I am amused that maybe you told or recommended them that they use or do something, and another person can come here and be able to sell them a tractor (...). We have no power of conviction (...), there comes a time that he/she says: ‘it is normal that you come and tell me this, and others come and tell me that’ (...)" [Original: “(…) Yo no sé si podemos convencerlos suficientemente, porque me hace gracia que a lo mejor tú les dices o recomiendas que utilicen o hagan cualquier cosa, y es capaz de venir un señor a venderles un tractor (…). No tenemos poder de convicción (…), llega un momento que dice: ‘ya es normal que vengas tú y me digas esto, y viene este y me diga lo otro’ (…)”]*

VG4: *"(...) It is something that is so implanted that it is not easy to say: ‘we must do this and this’ (...), I think there is a lack of more ways to propose it (...). I do not think that people are closed, I think maybe we do few apostolates (...)" [Original: “(…) Es algo que está tan implantado que no resulta fácil decir: ‘hay que hacer esto y esto’ (…), creo que faltan más formas de proponerlo (…). Yo no creo que la gente esté cerrada, creo que quizás hacemos pocos apostolados (…)”]*

As far as gender is concerned, female veterinarians indicated that farmers that have previously worked with them usually respect their professionalism, just as they respect that of a man. However, those farms that have not worked with women previously tend to value their work over time. In the same way, although there may be situations in which sexist dynamics persist, such as those involving physical effort, female veterinarians pointed out that farmers have more confidence in women to share issues of deeper personal aspects. Despite this, it did not stand out clearly in these interviews that the fact of being a woman or man made any difference in influencing the decision of the farmers in relation to the application of biosecurity measures.

As regards the ability of the farmer to influence the decision to apply biosecurity measures by other farmers, bars, pubs, or restaurants were stressed as a space where there is greater interaction among farmers, due to them being locations prone to engage in relaxed conversations and acquire knowledge (which may be reliable or not). Indeed, from the point of view of the farmers themselves, it was emphasized that they could provide information that may be incomplete if it is related to their own farms. In these places there are farmers who are capable of being vocal or leaders and influence others, although those who are considered as reference models are those farmers who are innovators or pioneers in certain areas, and who own large farms.

The relationship between farmers was not only limited to a collective space such as bars, pubs, or restaurants; in fact, the visits to other farms were emphasized by the veterinarians. These visits tend to have positive effects on the farmers through observations and a later reflection, which may lead to the application of biosecurity measures. In these visits the farmers can find out realities different from theirs, being totally disposed to its realization.

The events that occur in the neighbouring farms (proximity experiences) were highlighted as another relevant factor in the decision to apply biosecurity measures, for example, outbreaks of infectious diseases in other farms. In these cases, neighbouring farmers that have not been affected begin to deploy a series of actions to prevent the possible entry and spread of that infectious disease in their farm. This kind of learning was featured as one of the most important, since the unaffected farmers are placed in a scenario where that could happen, imagining their possible consequences. In the same way, this type of event might be used as an example by certain veterinarians to encourage their farmers to implement biosecurity measures and thus avoid experiencing similar situations. However, it should not be forgotten that each farmer has its own economic, social, cultural and political contexts. This means that they have their own factors that can affect their decision‐making process to implement biosecurity measures.

### 3.2 Internal world of the farmer

Individual factors of farmers can determine their perceptions regarding the feasibility of implementing biosecurity measures. Factors reported in the interviews as most relevant could be grouped in adaptation, pre‐disposition and individual experiences.

The ability of farmers to adapt to changes was associated with a greater capacity to progress. Despite this, the interviewees stressed that these changes are not always easy to carry out since farmers are usually people with habits, and therefore, they are not always prepared to face and tolerate these changes, a situation that generates their bewilderment and fear, especially when these changes are drastic.

The pre‐disposition of farmers to implement biosecurity measures was mentioned to be linked to their effectiveness and benefits. Resistance, carelessness or lack of interest could be generated if they do not see a return to their actions and feel difficulties in their performance. Some farmers believed that biosecurity measures could avoid disease risk and health problems, improving therefore their productivity due to an enhance in the health status of the herd. In this way, some farmers indicated that biosecurity was essential and that without it their farms would not work. The interviewees also perceived that biosecurity was important to not fear infectious diseases enabling themselves to focus in the improvement of other areas. On the other hand, there were participants who had not convinced that biosecurity measures could generate benefits. For example, some commented that there are other productive systems (e.g. swine) that have also faced health problems, although implementing several biosecurity measures. In the same way, they revealed that biosecurity was not their priority and preferred to invest in other areas and that those measures could be complicated to carry out without observable effects in the short term.

The pre‐disposition to implement biosecurity measures was not clearly linked to their financial situation. For example, there were farmers who were highly willing to invest in prevention but whose limitation was their economy, and other farmers who were not willing to invest in prevention since they prefer to invest to grow. Even though the advice of the veterinarian, some interviewees mentioned that farmers would invest on biosecurity depending on the profitability or the urgency of biosecurity measures.

Individual experiences of the farmers were reported as the factor with the greatest impact on farmers to implement biosecurity measures. For example, farmers who experienced outbreaks of infectious diseases on their farms attributable to not having good biosecurity had subsequently begun their implementation. In fact, it was expressed that if they had not experienced a similar negative situation, they would not have valued those measures. In addition, these experiences motivated farmers to seek more information, such as about infectious diseases, for better understanding.

Other factors, such as the training that farmers have, were also pointed out. Some farmers commented that they do not know the risk of certain external agents entering their farms, a situation that can perpetuate their risk dynamics. Another thing that stood out was that farmers usually demand information from their veterinarians; therefore, they have the willingness to learn. However, although farmers can receive training from veterinarians, they should learn issues pertinent to livestock as business (e.g. personal management) and on other professional areas:

VG3: *"(...) That the farmer has training and sees through training how important it is in his/her business to take biosecurity measures (...). Let's say we give them training on a day‐to‐day basis, whenever you go to visit them, you are advising them with training (...)" [Original: “(…) Que el ganadero tenga formación y vea a través de la formación lo importante que es en su negocio llevar medidas de bioseguridad (…). Digamos que la formación se la damos en el día a día, siempre que vas a visitarlo le estas asesorando con formación (…)”]*

VG8: *"(...) There is still a lot (...) in the training part (...). The farmer must receive training as a farmer, the farmer cannot receive training as a veterinarian, nor as an agronomist, because for that he/she would have to go to a university (...)" [Original: “(…) Todavía queda mucho (…) en la parte de formación (…). El ganadero tiene que recibir formación de ganadero, el ganadero no puede recibir formación de veterinario, ni de ingeniero agrónomo, porque para eso tendría que acudir a una universidad (…)”]*

### 3.3 Social dynamics (internal and external)

Dairy farms (farmers and veterinarians) are inserted in different social media that can generate different social dynamics, which can be internal or external.

The internal dynamics refer to intrinsic issues of the dairy farms. In this way, the participants mentioned that dairy farms have different characteristics from other productive systems such as swine, which can be a limitation to implement biosecurity measures. These characteristics, in the opinion of the people interviewed, might be linked to the tradition of certain farms, such as, the visits of people without a previous appointment. However, this tradition is currently undergoing major changes, for example, certain farms are evolving, in words of a veterinarian, ‘*from being an extension of the kitchen to being a business*’. On the other hand, the effect of pressure or social influence was also highlighted, which may be greater in rural contexts than in urban contexts, especially when there are events that generate alarm in the population, such as public health issues, which increases interest in biosecurity.

The external dynamics refer to issues specific to the social factors (inside and outside the dairy farms) and to the various degrees of social cohesion. In this sense, it was pointed out that inside the dairy farms there is a coordination between the farmers and their different veterinarians with a joint task. However, there was no perceived coordination outside, between private and official veterinarians, as well as with other sectors (universities, dairy industry, or laboratories). As regards the OVS, it was mentioned that farms and veterinarians that belong to the OVS are in parallel worlds, since the last ones only watch over the compliance of the protocols and they are not involved in the farms like private veterinarians. Furthermore, private veterinarians featured their role as intermediaries between the farmer and the OVS. It was also mentioned that pressure from private and official veterinarians drives farmers to implement biosecurity measures. The need for better coordination was stressed, with the involvement of all people who interact internally (farm workers, farmers or veterinarians) or externally (OVS, universities, dairy industry or laboratories) on the farms should direct their efforts in the same direction.

It is important to mention that although it is the farmer that does the training or resorts to certain sources of information, it is the farm workers who finally perform the actions. These workers have different types of profiles which can vary mainly by gender (men and women), age (20–55), nationality (national or foreign) and previous experience in farms (present or absent). This diversity of profiles was linked to the scarce availability of labour:

VG1: *"(...) Here there are young people, from 20 years olds to people over 55, women, national people, foreign people. They do not meet a profile, as you work more with protocols, you look for a worker who meets them and that’s it (...), they are farm workers (...)" [Original: “(…) Aquí hay desde gente joven de 20 años a gente mayor de 55, mujeres, gente nacional, gente extranjera. No cumple un perfil, como se trabaja más a protocolos, buscas un trabajador que los cumpla y listo (…), son operarios de granjas (…)”]*

FG5: *"(...) If I do not install facilities, no, to have them wrong. I'm tired of having things wrong, and it also gives a lot of work. And workforce is very limited, there is no workforce (...). Workforce is needed (...)" [Original: “(…) Si no hago instalaciones, no, para tenerlas mal. Estoy cansado de tener las cosas mal, y además da mucho trabajo. Y la mano de obra es muy escueta, no hay mano de obra (…). La mano de obra hace falta (…)”]*

### 3.4 Official veterinary services: bad policemen or necessary enemies?

Farmers and OVS may interact because of existing legislation. Public administrations force farmers to implement specific biosecurity measures that they probably would not do voluntarily unless they experienced certain complications. In fact, some participants pointed out that mandatory actions are important, unlike those of a voluntary nature, as they allow farmers to move forward to implement these measures. However, it was indicated that some regulations could be improved to make biosecurity measures feasible to implement.

Regardless of the obligatory nature and the feasibility of the biosecurity measures, some situations that can generate mistrust towards the public administration, and consequently compromise the credibility of their recommendations, were mentioned. The situations mentioned by the interviewees could be grouped into questionings about measures and comparative grievances.

In relation to the questionings about certain measures, which may vary depending on the infectious disease, the participants attributed negative consequences to the farms by applying compulsory vaccination programmes (collective experiences). For example, some participants reported several productive losses after vaccination against bluetongue, causing its use to be feared by farmers and not recommended by veterinarians. They also challenged the real importance of certain measures, such as the perimeter fences, which went from mandatory to voluntary. In fact, some participants pointed out that the OVS show contradictions as to whether to implement biosecurity measures. Likewise, the participants questioned certain situations in which the official veterinarians recommended fencing, but leaving the gates open in case a common road crossed the farm or dividing the farm with two fences in case a stream crossed the farm.

In relation to the comparative grievances, there were farmers who made comparisons with other farmers and other productive systems. Some farmers pointed out that all dairy farms should be under the same rules to be on equal terms, an issue that could be favourable for dairy production itself. Likewise, they commented that other productive systems (e.g. goats and sheep) should also be subject to the same rules. Farmers did not want them (OVS) to be more flexible or permissive with their farms, since they agree with them, but they demand minimums from all ruminants without exception.

All these factors, added to other experiences, have led to the farmers having a certain perception of the OVS. There are collective opinions that believe that public administrations do not understand the realities of farms and that they should know and have a closer contact with them to subsequently generate legislation that considers their realities since, in the opinion of the interviewees, they frequently create regulations complicated to perform (e.g. the perimeter fences). In addition, from their point of view, sometimes the official veterinarians can be very severe and apathetic, creating problems in the farms that previously did not exist. This situation leads to them being defined by some farmers as ‘*bad policemen*’, who only penalise, although there may be exceptions in that they believe that some are understanding and facilitators.

Some farmers were aware that the public administration just do their work and that this can favour their farms. In fact, some veterinarians used the term ‘*necessary enemies*’ to define the official veterinarians (OVS). In this way, farmers highlighted that public administrations are essential, although they might be slow in their management (bureaucracy), a situation that can be evidenced by their late responses, but that can play an essential role in the application of biosecurity measures in the farms. Some veterinarians commented that the penalties (e.g. fines) can lead to farmers implementing these measures, but also the incentives (e.g. subsidies) if they meet specific conditions. However, some farmers mentioned that the farms should not operate by incentives, but on their own account as a business without depending on them:

VC2: *"(...) The farmers’ perception is that the administration always tries to penalise, rather than advising or helping to solve the problem. They are people who, when they come to control routinely, or by surprise, an exploitation, always try to look only for the bad, that is their perception, it's like when the police stop you and you do not know why (...)" [Original: “(…) Su percepción es que la administración siempre intenta penalizar, más que asesorar o ayudar a solventar el problema. Son gente que cuando vienen a controlar de manera rutinaria, o por sorpresa, una explotación, siempre intentan buscar solo lo malo, esa es su percepción, es como cuando te para la policía y no sabes por qué (…)”]*

VC3: *"(...) The administration, in most of the farms, is conceived as the bad policeman (...), as a necessary enemy (...). Inspectors who have zero empathy (...), there is also someone who (...) is considered as an ally in the farm (...)" [Original: “(…) La administración, en la mayor parte de las granjas, es concebida como el policía malo (…), es un enemigo necesario (…). Inspectores que tienen cero empatía (…), también hay alguno que (…) se le considera como aliado en la granja (…)”]*

FG1: *"(...) People who are there do not understand much about what farming is, they should know more about this (...). They should generate other stuff that were related to each zone (...)" [Original: “(…) La gente que está allí no entiende mucho de lo que es una explotación, deberían saber más de lo que es una explotación (…). Deberían sacar otras cosas que fueran relacionadas a cada zona (…)”]*

FG2: *"(...) The relationship with the head of the health area is good, I think they have to do their job and they do it (...), what mistakes everyone has, but they are very understanding, and I think that they defend themselves in their area (...)" [Original: “(…) La relación con el responsable del área de sanidad es buena, yo pienso que tienen que cumplir su trabajo y lo hacen (…), que errores los tiene todo el mundo, pero sí que son muy comprensivos y pienso que se defienden en su área (…)”]*

### 3.5 Variables of time and space

Time and space available were two other factors that were highlighted as barriers for the application of biosecurity measures. On one hand, time was reported as a limitation for farmers and veterinarians to implement these measures and to conduct training or to resort to sources of information, such as visits to farmers. This was because the farmers usually perceive to have too many hours of work and the veterinarians usually work with defined times in each farm, depending on their profiles and demands, being able to cover (or not) these measures. And, on the other hand, the available space in the farms could also influence the implementation of biosecurity measures, since there are regulations that can restrict infrastructure constructions. For example, because of this, farmers might buy external animals since they cannot make their own replacements due to space limitations.

## 4 DISCUSSION

According to the ‘Animal Health Law’ (European Parliament & EU Council, 2016), the implementation of biosecurity measures at farm level is the responsibility of the farmers. Therefore, in the context where there are no policies that force farmers to implement biosecurity measures, and in a sector where the implementation of biosecurity measures is scarce (Sahlström et al., 2014; Sarrazin et al., 2014), developing strategies to motivate farmers is of paramount importance to achieve an improvement in biosecurity. Nevertheless, the development of such strategies should be based on an understanding of the different psychosocial factors influencing farmers’ decision‐making. The present study, to the best of our knowledge, is the first one that has attempted to do so in dairy farms in Spain.

One important factor that arose from the interviews was the influence of the private veterinarian. As previously described in other studies (e.g. Cardwell et al., 2016), veterinarians are considered to be the main source of information for farmers to learn about biosecurity and therefore their training and communication skills are highly relevant (Hamood, Chur‐Hansen, & McArthur, 2014; Ruston et al., 2016). In this sense, some researchers have pointed to the fact that veterinarians usually give more importance to their own knowledge than to the opinion of their clients (e.g. farmers), and therefore, they are paternalistic (Bard et al., 2017), which highlights the importance of establishing a dialogue with consensus between farmers and veterinarians (Kuster et al., 2015). In our study, the interviewed veterinarians emphasized that they see farmers as an equal and that they usually have a horizontal relationship, a situation that can facilitate an effective communication. However, some of them mentioned feeling uncomfortable to recommend biosecurity measures due to the possible reactions that the farmers may have (e.g. their fear that farmers believe that there may be a conflict of interest). Interestingly, this was not mentioned by any farmer. Therefore, the relationship between farmers and veterinarians could incorporate personal and professional aspects with transparent dialogues to be close and reliable without misunderstandings, helping to ensure that biosecurity measures can be internalised in a better way.

As for the veterinarian's profiles, the HDA veterinarians were identified as those that are mainly responsible for biosecurity, being consistent with their role played; however, there are still farms that scarcely implement biosecurity measures. This could be due to the existence of obstacles in their relationship, as was described in Sweden (Svensson, Alvåsena, Eldh, Frössling, & Lomander, 2018). According to these researchers, although the health management veterinarians are important and have a similar professional profile to those of the Spanish HDA veterinarians, farmers do not always carry out their suggestions because of difficulties in their relationships. Furthermore, in this study, these obstacles are not directly linked to the time that veterinarians spend on the farms, since they visit the farms with the same frequency of other veterinarians’ profiles (e.g. clinical and reproduction). Thus, it would be particularly interesting to look deeper into this profile, as their role is directly related to biosecurity measures, unlike other profiles that indirectly approach this issue.

Other factors were mentioned in relation to the pre‐disposition of providing advice to farmers about biosecurity, such as their lack of sufficient training in the topic, or the risk of developing fatigue in farmers due to their insistence. Further studies to look deeper into all the aspects related to the communication process between farmer and private veterinarians are, in our opinion, of paramount importance, as seems to play a central role in the implementation of biosecurity measures by farmers. Issues such as transparency of relations between farmer and veterinarians or the position that the veterinarian should have in front of the farmers, together with the necessary steps to achieve this, might be required before developing adequate motivation strategies.

Individual experiences were also highlighted to heavily influence the decision‐making process of the farmer to implement biosecurity measures. Interviewees mentioned increasing biosecurity in the event of a public health problem or an outbreak of a disease in a neighbouring farm (proximity experiences), as previously described in different studies (e.g. Hernández‐Jover, Taylor, Holyoake, & Dhand, 2012) but, interestingly, none of them (farmers nor veterinarians) linked biosecurity as a way to reduce the risk of disease transmission in the scenario of the introduction of an exotic disease in the country (e.g. foot and mouth disease). This could reflect that farmers and veterinarians have a lack of awareness about these diseases that may deserve further attention.

Moreover, collective experience may also play a role in the decision‐making process, giving rise to doubt about the effectiveness of some biosecurity measures carried by OVS or resistance to their implementation. As, for example, there are still farmers today that remember what happened with the vaccination against bluetongue (2006–2009) (Sok, Hogeveen, Elbers, & Oude, 2016), and it might not let the farmers fully trust in the public administration. This kind of experience is difficult to approach, since it has a repeated retrieval and feedback among the farmers (Roediger, Zaromb, & Butler, 2009), and it should be kept in mind when trying to reach the farmers. Therefore, the strategies to face these experiences must combine unified official discourses with transparency and awareness, which together could gradually have an impact on the farmers’ decision‐making.

On the other hand, the pre‐disposition of farmers to implement biosecurity measures was linked to the effectiveness and benefits of the measures, as reported in previous studies (e.g. Alarcon, Wieland, Mateus, & Dewberry, 2014), which is an issue difficult to demonstrate. However, the perception of benefits does not always maintain a univocal relationship with the perception of risks, since sometimes, if benefits are perceived, risks are avoided, while in others they do not (Valeeva, Van Asseldonk, & Backus, 2011). This was demonstrated in our study, as farmers had different opinions in relation to the benefits of implementing biosecurity (from people that considered it essential to more sceptic people). The development of tools and the spreading of the results showing the potential benefits of biosecurity are also recommended in order to improve their perception of the cost‐effectiveness of biosecurity.

This study was focused on farmers and veterinarians, but the farm workers also appeared in their answers, as they are the ones that implement the biosecurity measures in the field. Interviewees mentioned that farm workers are scarce nowadays, and often with a low level of training, which has forced some farms to replace them with milking robots. The reasons can be varied, for example, it may be due to the existence of high levels of stress due to working conditions (health and safety) or workloads (Chen & Holden, 2016; Lunner et al., 2013). Considering the importance of farm workers in the implementation of the biosecurity measures in the field, performing studies focused on this group are also highly recommended.

Although, as previously mentioned, the European ‘Animal Health Law’ attributes the farmer with the responsibility of implementing biosecurity measures. In Spain, there have been some attempts by regional and national governments to develop specific legislation to force farmers to implement some recommendations, which has generated many discussions. Biosecurity measures imposed by legislation usually generate a lot of debate, as described elsewhere (e.g. Oliveira, Anneberg, Voss, Sørensen, & Thomsen, 2018). The role that OVS should play in the implementation of biosecurity measures is subject to debate and might also deserve further studies. According to the responses of some interviewees, legislation is needed to safeguard dairy farms, although they should be accompanied by an understanding of all the people involved, as proposed by Brennan and Christley (2013). However, the legislation and their obligatory nature is a complex issue to approach, a situation that becomes even more complex when they intersect issues, such as awareness. Thus, it would be interesting to look into the effect of the obligatory in future theorized discussions from a sociological perspective.

As for the methodology used in this study, we decided to use a qualitative methodology (i.e. semi‐structured in‐depth interviews) which can be appropriate to investigate and look deeply into the different realities of people (Mason, 2006). Qualitative methods are based on interpretivism and constructivism paradigms (multiple realities), while quantitative research is mainly based on a positivist paradigm (only an objective reality) (Sale, Lohfeld, & Brazil, 2002). Therefore, the repeatability of qualitative studies can be lower than for quantitative studies, since it considers that all interviewees have unique and unrepeatable realities (Leppink, 2017). However, this technique is adequate to determine the different interpretations of reality from the opinion of each of the participants (Della, 2014), which can be influenced by various factors that may be difficult to perceive by us.

As regards the intrinsic flexibility of the semi‐structured in‐depth interviews, it has to be borne in mind that the questions were carried out differently with each of the participants, that is, their order and content were varied in relation to their development with each of the participants. For example, gender questions were asked only to the women interviewed at different times. The objective of the above was to be executed a fluent and spontaneous interview, where the participants could feel comfortable and free (Ryan, Coughlan, & Cronin, 2009). Nevertheless, there is a possibility that this may have affected their response to some degree, although this procedure is characteristic of this technique. In relation to the number of participants involved in the present study, although a saturation of the discourse (heterogeneous group) was reached, there is the possibility that other small variables could have arisen if we had carried out more interviews. This is mainly due to the magnitude of the various psychosocial factors that affect each of the contexts, which are not generalizable.

Results from our study highlight the need of promoting awareness as the key to the implementation of biosecurity measures, since they must be understood for true implementation. However, motivation strategies might also include other aspects, such as direct participation of farmers and monitoring of efforts by the cohesion of all the people involved over time. Nevertheless, the development of such strategies would benefit from a deeper understanding of some of the topics identified through this study by using other techniques, such as an ethnography (Naidoo, 2012) or focus groups. Therefore, it has highlighted the impact that qualitative studies such as these can have, which can guarantee a greater representativeness of the data if carried out together with quantitative studies. This study was not intended to look deeply into each of the various factors separately, but to describe a global panorama of those that may exist among different dairy farms, identifying the main psychosocial factors that influence farmers’ decision‐making.

## 5 CONCLUSION

The decision to comply with the existing regulations and suggestions on the implementation of biosecurity measures in dairy farms are influenced by various psychosocial factors. In this study, we have identified the main psychosocial factors (and their interactions) that influence dairy farmers’ decision‐making in Spain. These factors are related to the relationship between farmers and veterinarians, the feasibility of implementing these measures, and the influence of social dynamics and OVS, together with the available time and space. All these psychosocial factors were identified as factors that influence the attitude and behaviour of farmers to implement biosecurity measures. In this way, the farmers function as complex systems that have certain psychosocial factors, which in turn can interact in different ways according to their economic, social, cultural and political contexts (i.e. they are heterogeneous).

In addition, these farmers can interact with other systems (e.g. veterinarians). The veterinarians appeared to play an important role in the dairy farmers’ decision‐making to implement biosecurity. Therefore, all the aspects that can influence the communication between dairy farmers and veterinarians such as trust, level of training or fears to provide recommendations, might play an important role and may deserve a deeper study in order to provide future recommendations to improve biosecurity. However, all these system interactions (farmers and veterinarians) can be further complicated if we consider other systems (e.g. farm workers and OVS). Thus, other aspects such as the internal social dynamics of farm workers and the role that OVS and the compulsory should play in the improvement of biosecurity were also identified as issues which need further analysis. In this way, this research represents a starting point to develop future recommendations to improve the implementation of biosecurity measures.

## ETHICS STATEMENT

The present study was approved by the Ethics Committee of the UAB (CEEAH 4055), who also helped design the informed consent for the participants.

The informed consent was used to mainly to explain the objectives of the study and the conditions and guarantees of the participants. It was pointed out that the data would be confidential and analysed anonymously, that there would be no economic benefits to participate, and that the interview would be recorded by audio or text. The decision to participate in the study was totally voluntary, and if they wished they could stop and leave the interview at any time. In this way, the informed consent was signed by the interviewee and interviewer, with a copy for each one of them.
